# Intimate partner violence and physical health in England: Gender stratified analyses of a probability sample survey

**DOI:** 10.1177/17455057251326419

**Published:** 2025-03-25

**Authors:** Ladan Hashemi, Anastasia Fadeeva, Nadia Khan, Sally McManus

**Affiliations:** 1 Violence and Society Centre, City St George’s, University of London, London, UK; 2 School of Health and Psychological Sciences, City St George’s, University of London, London, UK; 3 National Centre for Social Research, London, UK

**Keywords:** domestic violence, intimate partner violence, IPV, physical health conditions, gender, men, women

## Abstract

**Background:**

Gender differences in the associated health outcomes of different forms of intimate partner violence (IPV) are understudied. The long-term effects of IPV on specific physical health conditions are also under-researched in comparison to the effects on general health and mental health.

**Objectives:**

To examine gender differences in the association between IPV and specific physical health conditions, accounting for differences in the types and number of types of IPV experienced.

**Design:**

We used data from the 2014 Adult Psychiatric Morbidity Survey, a cross-sectional survey using a stratified, multistage random sampling design to cover the household population of England aged 16 years and older.

**Methods:**

Descriptive and multivariable regression analyses of 4120 women and 2764 men who had ever had a partner. Lifetime IPV by types (physical, sexual, psychological, and economic), any lifetime and recent IPV, the number of IPV types experienced, and multiple chronic health conditions experienced over the past 12 months were included in the analyses.

**Results:**

Gender differences were observed in both the prevalence of IPV and associated health conditions. Women were more likely to experience any type and a higher number of IPV types than men. Women’s exposure to any lifetime and 12-month IPV were significantly associated with an increased likelihood of reporting 12 and 11 conditions, respectively, while men’s exposure to any lifetime and 12-month IPV were significantly associated with 4 and 1 conditions, respectively. Specific IPV types had varied health impacts, particularly among women. A cumulative association was evident for women but not for men.

**Conclusion:**

Healthcare systems need to be mobilised to address IPV as a priority health issue for the female population. Our findings highlight the need for gender-informed approaches in IPV intervention strategies and healthcare provision, emphasising the development of IPV-responsive healthcare systems and comprehensive IPV curricula in medical and health training.

## Plain Language Summary

Why was the study done?

Intimate partner violence (IPV) is a widespread global public health issue with serious and long-lasting consequences. While much research has focused on the mental health consequences of IPV, such as depression, PTSD, and substance misuse, there is limited evidence regarding its association with physical health. This study explored how different types and numbers of IPV are linked to specific physical health conditions and whether these associations differ between men and women.

What did the researchers do?

The researchers analysed data from the 2014 Adult Psychiatric Morbidity Survey in England, which included 4,120 women and 2,764 men who had ever had a partner. They examined the links between various types of IPV (physical, sexual, psychological, and economic) and a range of long-term health conditions.

What did the researchers find?

Women were more likely than men to experience IPV and a higher number of its types. There was evidence that women’s exposure to IPV was associated with a greater number of health conditions, and the more types of IPV they experienced, the worse their health outcomes. These associations were stronger for women than men and remained significant even after adjusting for socioeconomic factors and childhood abuse, highlighting the persistent relationship between IPV and women’s health.

What do the findings mean?

These findings suggest that IPV is a gendered issue, with stronger associations between IPV and physical health for women than for men. This may be because women are more likely to experience more and multiple types of IPV, more frequently, and more often with injury. Healthcare systems must recognise IPV as a priority issue, ensuring support is tailored to those affected. The study emphasises the need for gender-informed approaches in healthcare and IPV intervention strategies to improve health outcomes for both men and women.

## Introduction

Intimate partner violence (IPV) is an extensive global public health problem that affects individuals across diverse socio-cultural backgrounds and geographical regions.^1^ IPV is often defined as physical, sexual, emotional or psychological, economic violence/abuse or controlling behaviours occurring between former or current intimate partners.^1^ The World Health Organization (WHO) estimates that among women aged 15–49 the global lifetime prevalence of physical and/or sexual IPV is 27%, and past-year prevalence 13%.^2^ However, there is variation between countries, with lifetime prevalence ranging from 13% to 61% for physical IPV, 6% to 59% for sexual IPV, and 20% to 75% for psychological IPV.^3,4^ In 2014, the prevalence of physical IPV in the UK was among the highest of all EU Member States, with 28% of women reporting having experienced physical violence since the age of 15, second only to Denmark at 29%.^5^ By 2018, the estimated lifetime prevalence of physical or sexual IPV among ever-partnered women aged 15–59 in the UK remained high at 24%.^2^ For the year ending March 2023, the past-year prevalence of IPV among women aged 16 and older was estimated at 4%.^6^

Gender disparities are notable in experiences of IPV. While men also experience IPV, women encounter more frequent physical violence, more severe violence, higher rates of sexual IPV, increased coercive control, greater instances of injury, and heightened fear.^7–10^ The UK Office for National Statistics estimated that 2.1% of men aged 16+ experienced IPV in 2022–2023.^6^

IPV can have severe and long-lasting consequences including significant implications for the physical, mental, economic and social wellbeing of individuals and communities.^11,12^ The economic and social impact of IPV is substantial, with an estimated cost of £66 billion to the economy of England and Wales in 2017.^13^ A significant portion of this economic burden falls on the victims, followed by governmental expenses, particularly in relation to healthcare expenditure.^13–15^

Despite a growing body of evidence linking IPV to health conditions, significant research gaps remain. Research predominantly focuses on women or gender-neutral samples, with limited research investigating the experiences of men who have experienced IPV.^16,17^ This includes the lack of analysis of gender differences, which could provide valuable insights into the varying impacts and manifestations of IPV across genders.^18,19^ Moreover, research has been primarily focused on the mental health impacts of IPV, such as depression, posttraumatic stress disorder (PTSD), suicidal ideation, sleep disturbances, and substance abuse.^11,20,21^ However, there is a paucity of clear evidence regarding its effect on physical health.^18^ When physical health is addressed, the focus tends to be on health-related quality of life, general health, or acute impact such as injuries or homicide rather than specific chronic conditions.^18,19,22^ Research on chronic physical health conditions has primarily explored particular aspects of physical health, including chronic pain and gynaecological/obstetric conditions such as sexually transmitted diseases and reproductive health, but detailed investigations into other specific physical health conditions such as cardiovascular conditions and neurological symptoms and conditions are scarce or inconsistent.^18^ In some studies, researchers have had to aggregate data on specific physical health conditions into broader categories (e.g., experiencing at least one physical health condition) due to small sample sizes,^23^ further obscuring detailed insights.

Moreover, most research has focused only on physical and/or sexual IPV, often disregarding less visible forms such as psychological and economic IPV.^17,18,24,25^ This dearth of research poses a significant issue, as evidence indicates that these behaviours are prevalent in abusive relationships.^25,26^ Limited existing research on the link between non-physical forms of IPV and health outcomes has revealed their significant impacts, particularly on mental health indicators.^9,26,27^ However, their association with physical health remains even less explored. While different types of IPV tend to co-occur,^20,25,27,28^ research has typically treated them in isolation, overlooking the interplay between multiple forms of abuse and the fact that most victims (particularly women) experience more than one type of IPV simultaneously.^25,28,29^ Additionally, there is a lack of research examining the impacts of both recent and lifetime IPV on physical health outcomes.^23,30^

Other limitations pertain to the composition of the study samples. For example, some studies have recruited participants from IPV support or healthcare services rather than from population-based samples, which may limit the generalisability of their findings to the broader and more diverse population.^31^ Previous research on violence and health has also been limited by being restricted to people of working age, samples without upper age limits are needed to examine a range of health conditions.^32^

Moreover, there is a need to control for confounders such as various socioeconomic characteristics. While numerous studies have noted the importance of adjusting for childhood abuse,^31,33^ many fail to do so.

Utilising data from a representative probability sample in England, this study aimed to assess gender differences in the association between lifetime and recent (past 12-month) IPV exposure, disaggregated by type and cumulative exposure, and multiple specific health conditions including less studied conditions, while adjusting for various socioeconomic characteristics and childhood abuse.

## Methods

### Data and sample

We use data from the 2014 Adult Psychiatric Morbidity Survey (APMS), a cross-sectional survey that covers the household population of England aged 16 years and older, using a stratified, multistage random sampling design, based on the Small User Postcode Address File.^34^ As with most other UK government-funded general population surveys, inclusion criteria for the survey were being aged 16 years or older, being able to speak English, and living in a private household in England. Individuals living in temporary housing, those residing in institutional or communal establishments, and individuals who were sleeping rough were out of the scope of the sample. From the selected addresses, one adult was chosen per household. Fieldwork was conducted from May 2014 to September 2015, and, as with other government-funded UK surveys, verbal informed consent was obtained from all participants.

The resulting sample was representative of the England population aged older than 16 years, living in private households at the time of the survey.^34^

Interviews were done in people’s homes (or elsewhere, if preferred) by trained research interviewers, and averaged 1·5 hours. They involved computer-assisted personal interviewing, with some sensitive information collected through computer-assisted self-completion in which participants used the interviewer’s laptop. Interviews could be conducted at a location preferred by the participant, such as a public place or even their workplace if they preferred it over their home. Interviewers provided assistance when needed, such as reading out the self-completion questions for participants with sight impairments or literacy challenges. All participants were given helpline information, including details about domestic violence-related services. Further methodological details are published elsewhere.^34^ The achieved sample comprised 7546 individuals, a 57% response rate. Those who had never been in an intimate relationship were excluded from the current analyses (201 people). A further 461 participants were excluded as they did not respond to the self-completion part of the survey including the questions on experience of IPV, yielding an analytic sample of 6884. The secondary analyses were approved by the committee at City St George’s, University of London, that considers medium-risk applications (ETH21220–299). The authors assert that all procedures contributing to this work comply with the ethical standards of the relevant national and institutional committees on human experimentation and with the Helsinki Declaration of 1975, as revised in 2008.

## Measures

### Physical health conditions

In the face-to-face section of the interview, participants were asked whether they had experienced any of the listed 22 physical health conditions in the past 12 months. Due to small sample sizes, three conditions affecting the brain and nervous system – namely Dementia or Alzheimer’s, Epilepsy/Fits, and Stroke – were combined into a single variable for Neurological conditions. This adjustment resulted in a total of 19 condition categories being included in the analyses (see Supplemental Table 1 for a list of these conditions). Binary variables were generated to indicate whether participants had experienced each and any of the physical health conditions in the preceding year.

Additionally, participants were asked to assess their general health status. Responses to the question ‘How is your health in general?’ were dichotomised into ‘good general health,’ (including responses of ‘excellent’, ‘very good’, or ‘good,’) and ‘poor general health,’ (which included ‘fair’ or ‘poor’ responses).

### IPV

To assess IPV, we used responses to a series of questions about participants’ experience of physical and sexual violence and economic and psychological abuse from a current or former partner, asked in the self-completion section of the interview. The IPV questions were adapted from the Crime Survey for England and Wales, originally based on the Conflict Tactics Scale.^35^ All IPV questions were asked about in relation to the past 12 months and since the age of 16, which we refer to as 12-month IPV and lifetime IPV, respectively. Variables were derived for the lifetime experience of each IPV type, the experience of any lifetime IPV, the number of lifetime IPV types, and any IPV within the past 12 months. For specific question wording and response options, see Supplemental Table 1.

### Childhood abuse

Participants were asked whether they had experienced physical abuse (before age 18) or sexual abuse (before age 16). A combined binary variable was derived indicating any experience of abuse in childhood. For specific question wording and response options, see Supplemental Table 1.

### Demographic and socioeconomic characteristics

Participants self-reported gender (women, men), age (banded for analysis), and ethnicity (grouped into White British; White other; Black or Black British; Asian or Asian British; and mixed, multiple, or other), whether they were able to keep their home warm during winter (yes, no), whether they had been in serious debt in the past year (yes, no) and area-level deprivation (quintiled English Index of Multiple Deprivation scores^36^). These were used to describe sample characteristics and adjust for potential confounding in multivariable analyses. The selection of confounders was based on the availability of data in APMS and previous literature that establishes a link between socioeconomic factors, IPV, and health outcomes.^37–39^

### Statistical analysis

Analyses were conducted in Stata/SE 17.0.^40^ Survey weights were applied to account for sampling methods and non-responses using the Stata svyset survey command.

The full weighting strategy is described in the survey reports.^41^ Given differences in health outcomes by experience of IPV among men and women in previous studies, we undertook all analyses for women and men separately. Missing data including: ‘do not know’ and ‘refused’ were excluded from all analyses. Less than 1.5% of any variable had missing data.

Weighted proportions described the prevalence of each sociodemographic characteristic, exposure to each, any, and multiple lifetime IPV types, any 12-month IPV, each health condition, at least one physical health condition, and good general health, stratified by gender.

Chi-square tests were used to determine whether there were differences in the distribution of each sociodemographic, IPV variables, and health variable by gender (Table 1).

**Table 1.** Sample characteristics and IPV prevalence.

Characteristics	Men *n* (*W*%)^a^	Women *n* (*W*%)^a^	Gender difference *χ*² (*p*-value)^c^
Total	2764 (48.4)	4120 (51.6)	29.2 (<**0.001**)
Age group			
16–34	520 (30.2)	929 (29.9)	
35–54	899 (35.6)	1437 (34.0)	
55–70	988 (26.2)	1249 (26.2)	
75+	357 (8.0)	505 (9.9)	
Ethnicity			4.0 (0.264)
White (British and other white)	2531 (88.4)	3732 (88.7)	
Black/Black British	61 (2.8)	117 (3.4)	
Asian/Asian British	121 (6.5)	172 (5.5)	
Mixed, Multiple, Other	46 (2.2)	83 (2.4)	
Neighbourhood deprivation level^b^			13.2 (**0.001**)
Least deprived two quintiles	1225 (41.8)	1651 (39.6)	
Moderately deprived area	567 (19.8)	871 (20.7)	
Most deprived two quintiles	972 (38.3)	1598 (39.7)	
Serious debt in past year			2.7 (0.102)
Yes	202 (7.4)	345 (7.7)	
No	2551 (92.6)	3748 (92.3)	
Able to keep house warm during winter			6.9 (**0.009**)
Yes	2578 (93.7)	3759 (92.5)	
No	172 (6.3)	324 (7.5)	
Childhood abuse			1.2 (0.278)
Yes	497 (16.9)	784 (18.5)	
No	2238 (83.1)	3295 (81.5)	
IPV			
Lifetime physical IPV	275 (10.0)	879 (19.4)	151 (<**0.001**)
Lifetime sexual IPV	6 (0.2)	179 (3.8)	107.8 (p<**0.001**)
Lifetime psychological IPV	263 (9.2)	897 (20.3)	177.2 (<**0.001**)
Lifetime economic IPV	118 (3.7)	425 (8.7)	83.2 (<**0.001**)
Any lifetime IPV	457 (16.0)	1247 (27.8)	167.5 (<**0.001**)
Any 12-month IPV	98 (3.8)	315 (7.2)	49.3 (<**0.001**)
Lifetime IPV type count			245.4 (<**0.001**)
0 IPV	2307 (84.0)	2873 (72.2)	
1 IPV type	295 (10.8)	493 (11.6)	
2 IPV types	122 (3.8)	446 (10.1)	
3 or 4 IPV types	40 (1.4)	308 (6.1)	
Physical health conditions			
Cancer	76 (1.8)	71 (1.4)	8.3 (**0.004**)
Diabetes	249 (7.1)	219 (4.7)	35.6 (**0.001**)
Migraine	241 (9.4)	702 (17.4)	96.8 (**0.001**)
Neurological conditions	52 (1.6)	46 (0.9)	6.9 (**0.009**)
Cataract problem	466 (14.1)	713 (16.0)	0.2 (0.630)
Hearing/ear conditions	431 (12.6)	415 (8.5)	46.8 **(<0.001**)
Heart attack	84 (2.3)	73 (1.5)	11.9 (**0.001**)
High blood pressure	600 (16.7)	794 (17.1)	6.0 (**0.014**)
Bronchitis	70 (1.8)	112 (2.3)	0.2 (0.637)
Asthma	208 (7.3)	448 (10.1)	21.5 **(<0.001**)
Allergies	240 (9.2)	535 (13.0)	30.6 **(<0.001)**
Gastrointestinal conditions	189 (6.0)	352 (8.4)	6.6 (**0.010**)
Liver conditions	43 (1.3)	34 (0.7)	8.0 (**0.005**)
Bowel/colon problem	136 (4.5)	324 (7.3)	23.0 **(<0.001**)
Bladder conditions	158 (4.0)	243 (4.9)	0.1 (0.750)
Arthritis	394 (10.4)	876 (18.1)	54.0 **(<0.001**)
Musculoskeletal conditions	725 (24.6)	1182 (26.6)	5.0 (**0.025**)
Infectious disease	16 (0.6)	14 (0.3)	2.2 (0.140)
Skin conditions	298 (10.0)	499 (12.0)	2.8 (0.091)
Any chronic condition	915 (27.5)	1255 (27.5)	5.2 (**0.022**)
Good general health	2177 (82.5)	3284 (81.9)	(0.342)

Abbreviations: IPV, intimate partner violence.

a Weighted % are presented.

b Least deprived areas comprise the two least deprived quintiles and most deprived areas comprise the two most deprived quintiles, based on the ranking of area-level English Index of Multiple Deprivation scores.

c *p* values in bold indicate significance at the <.05 level.

A series of binary logistic regression models were conducted to calculate the odds of experiencing health outcomes for those exposed to different IPV types (compared with those who did not report experience of each type), any lifetime and any 12-month IPV, and multiple IPV types. Unadjusted (OR) and adjusted odds ratios (AOR) were reported separately for women (Table 2) and men (Table 3). Model 1 shows unadjusted logistic regression models for IPV as an exposure variable on each health condition as an outcome variable. Model 2 shows the association between each exposure and health conditions adjusted for sociodemographic factors. Model 3 includes the adjustments in Model 2 as well as controls for childhood experiences of physical and/or sexual abuse. Results are reported with 95% CIs and statistical significance set at a 2-sided *P* < .05. The STROBE cross-sectional reporting guidelines were consulted when preparing this article.^42^

**Table 2.** Association between exposure to lifetime physical, sexual, psychological, economic, any lifetime and 12-month IPV and current physical health conditions among women in England.

Health condition	Lifetime IPV								
	Physical IPV			Sexual IPV			Psychological IPV		
	Model 1 OR (95%CI)	Model 2 AOR (95%CI)	Model 3 AOR (95%CI)	Model 1 OR (95%CI)	Model 2 AOR (95%CI)	Model 3 AOR (95%CI)	Model 1 OR (95%CI)	Model 2 AOR (95%CI)	Model 3 AOR (95%CI)
Cancer	0.90 (0.49–1.64)	1.17 (0.62–2.21)	1.22 (0.65–2.32)	1.18 (0.40–3.55)	1.34 (0.44–4.08)	1.43 (0.46–4.51)	0.95 (0.53–1.71)	1.31 (0.71–2.40)	1.36 (0.73–2.52)
Diabetes	1.11 (0.77–1.60)	**1.51 (1.00**–**2.26)**	1.37 (0.90–2.07)	0.88 (0.43–1.80)	1.12 (0.54–2.34)	0.94 (0.44–2.01)	0.96 (0.66–1.40)	1.40 (0.91–2.15)	1.28 (0.84–1.97)
Migraine	**1.63 (1.31–2.02)**	**1.39 (1.11–1.73)**	1.24 (0.98–1.56)	**3.59 (2.49–5.18)**	**3.18 (2.19–4.62)**	**2.76 (1.90–4.01)**	**1.72 (1.39–2.12)**	**1.46 (1.18.1.82)**	**1.33 (1.06–1.66)**
Neurological conditions	1.12 (0.54–2.23)	1.18 (0.49–2.80)	1.00 (0.42–2.42)	2.45 (0.82–7.31)	2.38 (0.66–8.64)	1.94 (0.50–7.54)	1.22 (0.58–2.54)	1.33 (0.56–3.18)	1.18 (0.49–2.82)
Cataract/sight problem	1.07 (0.86–1.33)	1.21 (0.96–1.53)	1.16 (0.92–1.46)	**1.57 (1.03–2.38)**	**1.72 (1.10–2.68)**	**1.60 (1.04–2.47)**	1.03 (0.82–1.30)	1.22 (0.95–1.58)	1.17 (0.90–1.52)
Hearing/ear problem	0.92 (0.69–1.24)	1.16 (0.85–1.57)	1.03 (0.76–1.38)	1.16 (0.69–1.94)	1.34 (0.77–2.31)	1.12 (0.64–1.97)	0.97 (0.73–1.29)	1.29 (0.95–1.75)	1.17 (0.86–1.60)
Heart attack	1.36 (0.78–2.36)	**2.08 (1.12–3.87)**	**1.83 (1.00–3.37)**	1.25 (0.42–3.72)	1.36 (0.37–4.96)	1.07 (0.28–4.07)	1.23 (0.71–2.16)	**2.01 (1.13–3.57)**	**1.80 (1.03–3.15)**
High blood pressure	0.83 (0.67–1.01)	1.10 (0.87–1.38)	1.09 (0.86–1.38)	1.02 (0.66–1.56)	1.24 (0.77–2.02)	1.23 (0.76–2.00)	0.76 (0.61–0.95)	1.03 (0.81–1.31)	1.01 (0.79–1.29)
Bronchitis	1.41 (0.92–2.18)	**1.76 (1.10–2.81)**	1.44 (0.88–2.35)	0.63 (0.19–2.08)	0.68 (0.20–2.30)	0.51 (0.15–1.70)	1.37 (0.86–2.19)	**1.86 (1.13–3.07)**	1.63 (0.96–2.75)
Asthma	**1.67 (1.33–2.11)**	**1.61 (1.24–2.09)**	**1.52 (1.16–1.98)**	**2.17 (1.40–3.36)**	**2.07 (1.33–3.22)**	**1.90 (1.19–3.03)**	**1.71 (1.36–2.16)**	**1.62 (1.27–2.08)**	**1.56 (1.22–2.01)**
Allergies	**1.58 (1.27–1.98)**	**1.46 (1.16–1.85)**	**1.33 (1.04–1.70)**	**2.27 (1.50–3.44)**	**2.12 (1.38–3.26)**	**1.85 (1.19–2.88)**	**1.40 (1.11–1.77)**	**1.28 (1.00–1.62)**	1.17 (0.91–1.50)
Gastrointestinal conditions	**1.49 (1.10–2.00)**	**1.57 (1.14–2.16)**	**1.48 (1.06–2.06)**	1.68 (0.98–2.86)	1.65 (0.94–2.88)	1.48 (0.84–2.61)	**1.75 (1.33–2.31)**	**1.92 (1.41–2.62)**	**1.84 (1.34–2.52)**
Liver problem	**3.40 (1.63–7.08)**	**3.50 (1.67–7.31)**	**3.08 (1.40–6.78)**	**7.12 (2.70–18.7)**	**6.86 (2.59–18.17)**	**5.68 (1.82–17.7)**	**2.96 (1.40–6.25)**	**3.14 (1.46–6.71)**	**2.75 (1.28–5.90)**
Bowel/colon problem	**1.84 (1.43–2.37)**	**2.03 (1.53–2.68)**	**1.90 (1.42–2.56)**	**4.44 (2.86–6.91)**	**4.60 (2.88–7.35)**	**4.25 (2.66–6.78)**	**1.99 (1.54–2.57)**	**2.16 (1.63–2.86)**	**2.05 (1.54–2.72)**
Bladder problem	**1.65 (1.19–2.29)**	**1.95 (1.39–2.74)**	**1.74 (1.19–2.54)**	**2.64 (1.45–4.78)**	**2.81 (1.48–5.34)**	**2.33 (1.22–4.45)**	**1.75 (1.26–2.43)**	**2.14 (1.47–3.11)**	**1.93 (1.30–2.87)**
Arthritis	1.02 (0.84–1.23)	**1.46 (1.16–1.85)**	**1.33 (1.05–1.70)**	1.13 (0.75–1.70)	1.37 (0.87–2.16)	1.17 (0.74–1.86)	0.95 (0.77–1.18)	**1.50 (1.16–1.94)**	**1.37 (1.06–1.78)**
Musculoskeletal problem	**1.50 (1.26–1.78)**	**1.55 (1.29–1.87)**	**1.43 (1.18–1.74)**	**1.89 (1.33–2.69)**	**1.89 (1.28–2.81)**	**1.66 (1.11–2.47)**	**1.34 (1.12–1.61)**	**1.42 (1.17–1.73)**	**1.32 (1.08–1.62)**
Infectious disease	**4.52 (1.40–14.57)**	**4.40 (1.23–15.7)**	**4.62 (1.19–17.92)**	1.05 (0.13–8.62)	0.96 (0.12–7.59)	0.90 (0.09–8.75)	**4.69 (1.41–15.6)**	**5.46 (1.48–20.1)**	**5.57 (1.42–21.9)**
Skin problem	**1.73 (1.36–2.20)**	**1.54 (1.20–1.98)**	**1.39 (1.08–1.80)**	**1.88 (1.21–2.90)**	**1.66 (1.05–2.63)**	1.39 (0.86–2.23)	**1.65 (1.31–2.09)**	**1.47 (1.15–1.88)**	**1.34 (1.05–1.72)**
Any chronic condition	1.05 (0.88–1.25)	**1.27 (1.03–1.57)**	**1.24 (1.00–1.53)**	1.29 (0.89–1.86)	1.46 (0.99–2.16)	1.40 (0.93–2.11)	1.06 (0.89–1.27)	**1.33 (1.09–1.64)**	**1.31 (1.06–1.60)**
Good general health	**0.57 (0.48–0.68)**	**0.53 (0.44–0.65)**	**0.59 (0.48–0.72)**	**0.43 (0.30–0.62)**	**0.42 (0.29–0.62)**	**0.49 (0.33–0.72)**	**0.55 (0.46–0.67)**	**0.49 (0.39–0.60)**	**0.53 (0.42–0.66)**
Health condition	Lifetime IPV						12-month IPV		
	Economic IPV			Any Lifetime IPV			Any 12-month IPV		
	Model 1 OR (95%CI)	Model 2 AOR (95%CI)	Model 3 AOR (95%CI)	Model 1 OR (95%CI)	Model 2 AOR (95%CI)	Model 3 AOR (95%CI)	Model 1 OR (95%CI)	Model 2 AOR (95%CI)	Model 3 AOR (95%CI)
Cancer	1.30 (0.63–2.70)	1.33 (0.64–2.75)	1.38 (0.68–2.82)	0.92 (0.54–1.57)	1.16 (0.66–2.04)	1.21 (0.69–2.13)	0.97 (0.40–2.35)	1.51 (0.62–3.69)	1.58 (0.64–3.92)
Diabetes	1.17 (0.74–1.83)	1.09 (0.69–1.73)	1.01 (0.63–1.62)	1.03 (0.74–1.42)	1.38 (0.96–1.99)	1.26 (0.87–1.82)	0.56 (0.27–1.18)	0.90 (0.42–1.93)	0.78 (0.37–1.66)
Migraine	**2.00 (1.52–2.63)**	**1.77 (1.33–2.35)**	**1.60 (1.20–2.15)**	**1.71 (1.41–2.07)**	**1.48 (1.21–1.80)**	**1.33 (1.09–1.64)**	**2.45 (1.85–3.24)**	**2.15 (1.61–2.87)**	**1.92 (1.43–2.58)**
Neurological conditions	1.22 (0.46–3.27)	1.14 (0.39–3.31)	1.02 (0.36–2.91)	1.15 (0.61–2.19)	1.31 (0.62–2.76)	1.18 (0.54–2.59)	1.31 (0.44–3.86)	1.60 (0.42–6.02)	1.37 (0.34–5.57)
Cataract/sight problem	1.27 (0.94–1.71)	1.24 (0.90–1.71)	1.20 (0.87–1.66)	1.12 (0.92–1.37)	**1.28 (1.03–1.60)**	1.23 (0.98–1.54)	1.05 (0.74–1.50)	1.35 (0.93–1.94)	1.26 (0.88–1.81)
Hearing/ear problem	1.21 (0.80–1.81)	1.10 (0.70–1.72)	1.01 (0.64–1.59)	1.02 (0.79–1.32)	1.30 (0.98–1.71)	1.17 (0.88–1.55)	1.11 (0.72–1.70)	**1.75 (1.10–2.79)**	1.54 (0.95–2.48)
Heart attack	1.20 (0.53–2.74)	1.12 (0.49–2.57)	1.00 (0.44–2.27)	1.12 (0.67–1.87)	1.62 (0.93–2.81)	1.41 (0.81–2.46)	0.41 (0.12–1.44)	0.62 (0.13–3.01)	0.48 (0.10–2.34)
High blood pressure	**1.40 (1.07–1.84)**	**1.41 (1.04–1.91)**	**1.37 (1.00–1.86)**	0.85 (0.70–1.03)	1.11 (0.90–1.38)	1.09 (0.88–1.36)	0.92 (0.64–1.31)	**1.61 (1.08–2.40)**	**1.60 (1.06–2.43)**
Bronchitis	1.49 (0.84–2.63)	1.38 (0.75–2.53)	1.20 (0.63–2.28)	**1.47 (1.00–2.19)**	**1.85 (1.20–2.85)**	1.58 (0.98–2.57)	0.53 (0.21–1.33)	0.79 (0.30–2.09)	0.62 (0.23–1.69)
Asthma	**2.02 (1.50–2.72)**	**1.80 (1.31–2.47)**	**1.73 (1.25–2.40)**	**1.65 (1.34–2.04)**	**1.61 (1.28–2.03)**	**1.54 (1.22–1.96)**	**1.56 (1.09–2.23)**	**1.54 (1.06–2.25)**	1.43 (0.97–2.10)
Allergies	**1.51 (1.15–1.98)**	**1.40 (1.05–1.87)**	1.25 (0.93–1.69)	**1.58 (1.28–1.95)**	**1.47 (1.18–1.84)**	**1.34 (1.06–1.68)**	**2.11 (1.54–2.88)**	**2.02 (1.45–2.80)**	**1.83 (1.31–2.56)**
Gastrointestinal conditions	**2.07 (1.47–2.92)**	**1.92 (1.35–2.75)**	**1.84 (1.28–2.63)**	**1.79 (1.38–2.32)**	**1.91 (1.45–2.53)**	**1.85 (1.39–2.46)**	1.45 (0.98–2.16)	**1.59 (1.05–2.43)**	1.46 (0.96–2.21)
Liver conditions	2.53 (0.92–6.92)	2.19 (0.72–0.67)	1.92 (0.61–6.01)	**2.81 (1.36–5.80)**	**2.84 (1.39–5.81)**	**2.49 (1.23–5.04)**	**6.43 (2.84–14.5)**	**7.48 (3.31–16.9)**	**6.57 (2.88–15.01)**
Bowel/colon conditions	**1.94 (1.37–2.74)**	**1.92 (1.33–2.78)**	**1.76 (1.21–2.58)**	**1.93 (1.50–2.50)**	**2.08 (1.57–2.74)**	**1.96 (1.48–2.60)**	**2.58 (1.75–3.79)**	**3.10 (2.07–4.66)**	**2.91 (1.94–4.35)**
Bladder conditions	1.24 (0.82–1.88)	1.16 (0.75–1.79)	1.01 (0.65–1.59)	**1.88 (1.41–2.53)**	**2.22 (1.61–3.06)**	**2.00 (1.41–2.83)**	**2.16 (1.35–3.45)**	**2.95 (1.78–4.87)**	**2.59 (1.55–4.31)**
Arthritis	**1.63 (1.24–2.15)**	**1.65 (1.19–2.30)**	**1.50 (1.07–2.10)**	0.97 (0.80–1.17)	**1.34 (1.07–1.68)**	1.21 (0.95–1.54)	0.76 (053–1.09)	1.39 (0.92–2.10)	1.23 (0.81–1.87)
Musculoskeletal conditions	**1.41 (1.11–1.79)**	**1.28 (1.00–1.64)**	1.17 (0.90–1.51)	**1.44 (1.23–1.69)**	**1.50 (1.27–1.78)**	**1.39 (1.16–1.65)**	**1.51 (1.16–1.97)**	**1.81 (1.36–2.42)**	**1.64 (1.23–2.20)**
Infectious disease	**5.85 (1.53–22.4)**	**5.49 (1.16–25.95)**	**5.55 (1.08–28.6)**	**3.34 (1.04–10.1)**	3.33 (0.96–11.5)	3.42 (0.91–12.86)	1.63 (0.33–8.02)	1.71 (0.39–7.48)	1.66 (0.39–7.04)
Skin problem	**1.90 (1.39–2.61)**	**1.76 (1.26–2.45)**	**1.59 (1.13–2.25)**	**1.83 (1.49–2.24)**	**1.67 (1.34–2.08)**	**1.52 (1.21–1.91)**	**1.87 (1.33–2.63)**	**1.65 (1.16–2.36)**	**1.46 (1.02–2.10)**
Any chronic condition	**1.60 (1.27–2.01)**	**1.55 (1.21–1.99)**	**1.50 (1.16–1.93)**	1.07 (0.91–1.26)	**1.31 (1.09–1.58)**	**1.27 (1.05–1.53)**	1.06 (0.78–1.42)	**1.51 (1.10–2.09)**	**1.46 (1.05–2.03)**
Good general health	**0.40 (0.31–0.51)**	**0.46 (0.36–0.61)**	**0.50 (0.38–0.66)**	**0.62 (0.52–0.73)**	**0.56 (0.47–0.68)**	**0.61 (0.50–0.77)**	**0.59 (0.44–0.78)**	**0.49 (0.36–0.67)**	**0.55 (0.40–0.75)**

Abbreviations: AOR, adjusted odds ratio; CI, confidence interval; IPV, intimate partner violence; OR, odds ratio.

Model 1: unadjusted odds ratio.

Model 2: odds ratio adjusted for age, ethnicity, whether one can keep home warm in winter, any serious debt, and area deprivation level.

Model 3: odds ratios adjusted for variables in model 2+ childhood physical or sexual abuse.

Bold figures in the table indicate 95% CI significance at a two-sided *p* < 0.05.

**Table 3.** Association between exposure to lifetime physical, sexual, psychological, economic, any lifetime and 12-month IPV and current physical health conditions among men in England.

Health condition	Lifetime IPV								
	Physical IPV			Sexual IPV			Psychological IPV		
	Model 1 OR (95%CI)	Model 2 AOR (95%CI)	Model 3 AOR (95%CI)	Model 1 OR (95%CI)	Model 2 AOR (95%CI)	Model 3 AOR (95%CI)	Model 1 OR (95%CI)	Model 2 AOR (95%CI)	Model 3 AOR (95%CI)
Cancer	0.39 (0.11–1.35)	0.65 (0.25–1.69)	0.76 (0.28–2.02)	NO	NO	NO	0.18 (0.04–0.76)	0.24 (0.06–1.05)	0.28 (0.06–1.29)
Diabetes	0.70 (0.41–1.21)	0.93 (0.53–1.64)	0.91 (0.52–1.60)	4.79 (0.76–31.12)	1.39 (0.12–16.20)	1.27 (0.11–15.16)	1.03 (0.66–1.62)	1.24 (0.74–2.06)	1.20 (0.72–1.99)
Migraine	**1.88 (1.25–2.84)**	1.43 (0.93–2.20)	1.40 (0.91–2.15)	NO	NO	NO	**2.11 (1.41–3.15)**	**1.66 (1.10–2.48)**	**1.62 (1.07–2.45)**
Neurological conditions	1.46 (0.57–3.73)	1.38 (0.49–3.91)	1.24 (0.39–3.93)	**17.33 (2.73–110.0)**	**21.53 (4.19–110.7)**	**15.69 (2.95–83.52)**	**2.23 (1.03–4.81)**	1.80 (0.73–4.44)	1.63 (0.63–4.20)
Cataract/sight conditions	0.68 (0.45–1.02)	0.70 (0.46–1.06)	**0.61 (0.40–0.94)**	0.79 (0.08–7.54)	0.91 (0.10–8.70)	0.69 (0.07–6.36)	1.18 (0.82–1.69)	1.23 (0.84–1.82)	1.11 (0.75–1.64)
Hearing/ear problem	0.54 (0.33–0.89)	0.72 (0.44–1.19)	0.66 (0.40–1.11)	5.41 (0.80–37.68)	8.30 (0.13–521.02)	7.48 (0.13–441.08)	0.87 (0.57–1.33)	1.12 (0.70–1.78)	1.07 (0.66–1.72)
Heart attack	1.11 (0.47–2.65)	1.22 (0.48–3.07)	1.13 (0.43–2.93)	5.64 (0.57–55.35)	5.83 (0.54–63.27)	4.75 (0.43–52.43)	1.20 (0.59–2.45)	1.18 (0.57–2.46)	1.03 (0.45–2.40)
High blood pressure	0.90 (0.62–1.33)	1.17 (0.77–1.79)	1.08 (0.71–1.65)	1.80 (0.29–11.31)	0.59 (0.06–5.68)	0.46 (0.05–4.43)	0.84 (0.58–1.22)	1.00 (0.66–1.50)	0.89 (0.58–1.36)
Bronchitis	1.06 (0.41–2.76)	1.27 (0.43–3.72)	1.13 (0.39–3.30)	4.85 (0.51–46.71)	2.64 (0.13–52.38)	1.90 (0.08–45.31)	1.26 (0.55–2.88)	1.33 (0.48–3.64)	1.17 (0.43–3.19)
Asthma	1.20 (0.76–1.90)	1.11 (0.70–1.77)	1.04 (0.65–1.66)	NO	NO	NO	1.49 (0.93–2.39)	1.43 (0.88–2.34)	1.34 (0.80–2.24)
Allergies	1.53 (0.97–2.39)	1.44 (0.91–2.28)	1.36 (0.86–2.14)	NO	NO	NO	1.44 (0.90–2.28)	1.46 (0.89–2.41)	1.39 (0.84–2.33)
Gastrointestinal conditions	1.48 (0.96–2.27)	1.55 (0.97–2.50)	1.46 (0.89–2.38)	NO	NO	NO	1.47 (0.91–2.39)	1.52 (0.94–2.45)	1.38 (0.83–2.29)
Liver problem	**2.39 (1.01–5.68)**	1.90 (0.85–4.23)	1.81 (0.82–3.98)	NO	NO	NO	**3.16 (1.34–7.49)**	2.43 (0.93–6.33)	2.34 (0.84–6.51)
Bowel/colon problem	**1.74 (1.04–2.90)**	**1.96 (1.14–3.35)**	1.66 (0.95–2.91)	NO	NO	NO	**2.26 (1.35–3.76)**	**2.73 (1.56–4.78)**	**2.27 (1.25–4.12)**
Bladder problem	0.76 (0.39–1.47)	1.04 (0.54–2.02)	0.89 (0.45–1.76)	**12.77 (1.90–85.96)**	**17.30 (3.07–97.44)**	**10.53 (2.09–53.09)**	1.51 (0.88–2.59)	**2.15 (1.21–3.82)**	1.77 (0.98–3.20)
Arthritis	1.11 (0.75–1.63)	1.50 (0.98–2.30)	1.47 (0.96–2.26)	5.29 (0.81–34.49)	2.94 (0.28–31.27)	2.58 (0.25–27.03)	1.35 (0.93–1.96)	**1.80 (1.18–2.76)**	**1.74 (1.14–2.66)**
Musculoskeletal problem	1.19 (0.88–1.61)	1.14 (0.84–1.55)	1.06 (0.77–1.45)	0.76 (0.12–4.65)	0.94 (0.16–5.42)	0.76 (0.12–4.59)	**1.80 (1.33–2.43)**	**1.64 (1.21–2.22)**	**1.56 (1.14–2.13)**
Infectious disease	1.53 (0.47–5.04)	1.65 (0.56–4.85)	1.41 (0.49–4.12)	NO	NO	NO	**3.48 (1.02–11.84)**	**4.15 (1.87–9.19)**	**3.69 (1.65–8.25)**
Skin problem	1.08 (0.68–1.70)	1.08 (0.67–1.74)	1.05 (0.64–1.70)	NO	NO	NO	**1.97 (1.32–2.94)**	**2.12 (1.40–3.22)**	**2.12 (1.37–3.30)**
Any chronic condition	0.94 (0.69–1.28)	1.10 (0.80–1.53)	1.04 (0.75–1.44)	0.95 (0.15–5.99)	0.30 (0.03–2.88)	0.24 (0.02–2.30)	0.92 (0.68–1.24)	1.00 (0.71–1.41)	0.92 (0.65–1.31)
Good general health	0.85 (0.61–1.18)	0.85 (0.60–1.20)	0.92 (0.64–1.31)	0.75 (0.12–4.59)	0.72 (0.15–3.44)	0.91 (0.19–4.21)	**0.51 (0.38–0.68)**	**0.49 (0.39–0.60)**	**0.53 (0.42–0.66)**
Health condition	Lifetime IPV						12-month IPV		
	Economic IPV			Any IPV			Any IPV		
	Model 1 OR (95%CI)	Model 2 AOR (95%CI)	Model 3 AOR (95%CI)	Model 1 OR (95%CI)	Model 2 AOR (95%CI)	Model 3 AOR (95%CI)	Model 1 OR (95%CI)	Model 2 AOR (95%CI)	Model 3 AOR (95%CI)
Cancer	0.70 (0.15–3.27)	0.79 (0.16–3.91)	0.88 (0.18–4.38)	0.42 (0.17–1.07)	0.65 (0.28–1.51)	0.77 (0.32–1.87)	0.43 (0.05–3.43)	1.16 (0.12–11.20)	1.30 (0.13–13.00)
Diabetes	0.87 (0.43–1.75)	0.66 (0.31–1.43)	0.65 (0.30–1.41)	0.69 (0.47–1.03)	0.85 (0.55–1.31)	0.84 (0.54–1.30)	0.61 (0.30–1.24)	0.83 (0.37–1.86)	0.82 (0.37–1.83)
Migraine	**2.24 (1.32–3.78)**	**1.77 (1.02–3.09)**	1.70 (0.96–3.03)	**1.89 (1.34–2.67)**	**1.51 (1.06–2.16)**	**1.48 (1.02–2.13)**	1.24 (0.65–2.35)	0.97 (0.51–1.86)	0.91 (0.46–1.77)
Neurological conditions	**4.07 (1.71–9.70**)	**3.31 (1.27–8.68)**	**3.10 (1.12–8.63)**	**1.99 (1.00–3.95)**	1.87 (0.80–4.38)	1.75 (0.70–4.41)	1.60 (0.73–3.54)	1.48 (0.56–3.91)	1.27 (0.47–3.40)
Cataract/sight conditions	1.52 (0.93–2.50)	1.27 (0.75–2.15)	1.19 (0.70–2.02)	0.90 (0.66–1.21)	0.98 (0.71–1.33)	0.88 (0.63–1.21)	0.83 (0.43–1.59)	1.16 (0.60–2.23)	1.04 (0.54–1.99)
Hearing/ear conditions	0.93 (0.52–1.68)	0.96 (0.51–1.81)	0.94 (0.50–1.75)	0.78 (0.55–1.10)	1.02 (0.71–1.46)	0.97 (0.66–1.42)	0.44 (0.19–1.05)	0.81 (0.32–2.06)	0.78 (0.31–1.95)
Heart attack	1.02 (0.34–3.02)	0.86 (0.29–2.59)	080 (0.25–2.50)	0.99 (0.53–1.85)	1.11 (0.56–2.20)	1.01 (0.49–2.07)	1.33 (0.39–4.54)	2.17 (0.49–9.57)	1.94 (0.44–8.47)
High blood pressure	1.24 (0.76–2.04)	1.13 (0.65–1.95)	1.06 (0.61–1.84)	0.89 (0.67–1.19)	1.17 (0.86–1.59)	1.07 (0.78–1.46)	0.62 (0.32–1.22)	1.08 (0.51–2.27)	0.96 (0.46–2.00)
Bronchitis	0.65 (0.14–2.92)	0.50 (0.09–2.64)	0.44 (0.08–2.51)	0.83 (0.37–1.84)	0.92 (0.36–2.35)	0.81 (0.31–2.11)	0.58 (0.13–2.62)	0.80 (0.13–4.78)	0.70 (0.11–4.47)
Asthma	1.60 (0.84–3.04)	1.47 (0.78–2.78)	1.38 (0.73–2.61)	1.30 (0.87–1.94)	1.24 (0.81–1.89)	1.18 (0.77–1.81)	1.50 (0.66–3.42)	1.52 (0.66–3.52)	1.42 (0.60–3.36)
Allergies	1.72 (0.96–3.08)	**1.84 (1.01–3.35)**	1.78 (0.98–3.26)	1.37 (0.95–1.98)	1.36 (0.92–1.99)	1.32 (0.90–1.94)	0.73 (0.33–1.62)	0.70 (0.30–1.60)	0.65 (0.28–1.50)
Gastrointestinal conditions	1.45 (0.72–2.91)	1.29 (0.63–2.63)	1.19 (0.57–2.50)	1.36 (0.92–2.02)	1.43 (0.94–2.17)	1.32 (0.85–2.05)	1.43 (0.65–3.15)	1.86 (0.81–4.24)	1.67 (0.71–3.97)
Liver conditions	NO	NO	NO	1.98 (0.91–4.32)	1.64 (0.72–3.71)	1.64 (0.69–3.91)	0.80 (0.18–3.53)	0.73 (0.16–3.38)	0.68 (0.14–3.19)
Bowel/colon conditions	**2.83 (1.43–5.62)**	**2.62 (1.27–5.40)**	**2.29 (1.06–4.93)**	1.52 (0.96–2.41)	**1.77 (1.09–2.87)**	1.50 (0.91–2.47)	0.81 (0.31–2.16)	1.17 (0.43–3.22)	0.94 (0.33–2.68)
Bladder conditions	0.75 (0.29–1.97)	0.80 (0.30–2.18)	0.69 (0.25–1.91)	0.87 (0.53–1.43)	1.19 (0.71–1.99)	0.97 (0.57–1.65)	1.02 (0.40–2.59)	1.94 (0.70–5.39)	1.61 (0.60–4.33)
Arthritis	0.89 (0.50–1.60)	0.76 (0.39–1.47)	0.73 (0.38–1.42)	0.96 (0.70–1.31)	1.26 (0.89–1.77)	1.21 (0.86–1.71)	0.70 (0.33–1.45)	1.19 (0.51–2.75)	1.12 (0.48–2.61)
Musculoskeletal conditions	**1.80 (1.20–2.71)**	1.52 (0.99–2.33)	1.46 (0.94–2.26)	**1.46 (1.15–1.85)**	**1.39 (1.09–1.77)**	**1.32 (1.03–1.70)**	1.16 (0.70–1.92)	1.20 (0.73–1.97)	1.11 (0.67–1.85)
Infectious disease	NO	NO	NO	2.16 (0.68–6.88)	**2.47 (1.10–5.55)**	2.17 (0.99–4.78)	**5.76 (1.36–24.44)**	**6.37 (2.03–20.00)**	**5.54 (1.78–17.28)**
Skin conditions	1.42 (0.79–2.55)	1.36 (0.75–2.49)	1.34 (0.73–2.48)	1.31 (0.92–1.87)	1.36 (0.95–1.95)	1.36 (0.93–1.98)	0.41 (0.16–1.06)	0.46 (0.18–1.20)	0.44 (0.17–1.16)
Any chronic condition	1.21 (0.79–1.85)	1.04 (0.65–1.67)	0.99 (0.61–1.58)	0.90 (0.71–1.15)	1.06 (0.81–1.39)	1.00 (0.76–1.32)	0.78 (0.45–1.34)	1.14 (0.62–2.13)	1.06 (0.57–1.97)
Good general health	**0.57 (0.37–0.89)**	**0.46 (0.36–0.61)**	**0.50 (0.38–0.66)**	**0.74 (0.57–0.96)**	**0.73 (0.55–0.96)**	0.76 (0.57–1.02)	0.84 (0.52–1.38)	0.68 (0.39–1.19)	0.74 (0.41–1.32)

Abbreviations: AOR, adjusted odds ratio; CI, confidence interval; IPV, intimate partner violence; OR, odds ratio, NO, no observation.

Model 1: unadjusted odds ratio.

Model 2: odds ratio adjusted for age, ethnicity, whether one can keep home warm in winter, any debt, and area deprivation level.

Model 3: odds ratios adjusted for variables in model 2+ childhood physical or sexual abuse.

Bold figures in the table indicate 95% CI significance at a two-sided *p* < 0.05.

## Results

The sample comprised 4120 ever-partnered women with a mean age of 52.97 years (*SD* = 18.27) and 2764 ever-partnered men with a mean age of 51.13 years (*SD* = 18.27). Men and women shared similar demographics in this representative sample, with some key differences. Women were slightly more likely to be in the oldest age group (9.9% of women and 8.0% of men were aged 75 or more) and to face financial strain. Specifically, 7.5% of women and 6.3% of men were unable to keep their homes warm in winter, and 39.7% of women and 38.3% of men lived in the most deprived neighbourhoods.

Women were more likely to experience any form of lifetime IPV, as well as each specific form of lifetime IPV compared to men. Experience of at least one form of lifetime IPV was reported by 27.8% of women and 16.0% of men. Regarding specific forms, 19.4% of women and 9.2% of men experienced physical IPV, 3.8% of women and 0.1% of men experienced sexual IPV, 20.3% of women and 9.2% of men experienced psychological IPV, and 8.7% of women and 3.7% of men experienced economic IPV. Women were also more likely to experience IPV in the past 12 months (7.2%) than men (3.8%).

Experience of multiple forms of IPV (more than one form) was reported by 16.2% of women and 5.2% of men.

Irrespective of IPV exposure, musculoskeletal conditions were the most common health condition reported, affecting 26.6% of women and 24.6% of men. Women were more likely than men to report experiencing migraines, high blood pressure, asthma, allergies, arthritis, musculoskeletal, gastrointestinal conditions, and bowel or colon conditions. Conversely, six health conditions, including cancer, diabetes, neurological conditions, heart attacks, hearing and liver conditions, were more common among men than women. The experience of at least one health condition was reported by 27.5% of both genders. No gender differences were found in the prevalence of good general health.

### Any lifetime IPV

After adjusting for socioeconomic factors, women who experienced any type of IPV during their lifetime had greater odds of having at least one health condition than women who did not experience any lifetime IPV (AOR: 1.31, 95% CI: 1.09–1.58). Exposure to any lifetime IPV was also associated with lower odds of experiencing good general health among women (AOR: 0.61, 95% CI: 0.50–0.77). Further adjustment for childhood abuse slightly attenuated the odds ratios, but they remained significant.

Regarding the association between exposure to any (at least one type of) lifetime IPV and specific health conditions, after adjusting for socioeconomic factors, women exposed to at least one type of IPV were more likely to experience 12 out of 19 studied health conditions than those with no IPV exposure. These conditions included migraine, bronchitis, asthma, allergies, arthritis, gastrointestinal conditions, and sight, liver, bowel/colon, bladder, musculoskeletal, and skin conditions. The adjusted odds ratios (AORs) ranged from 1.28 (1.03–1.60) for sight conditions to 2.84 (1.39–5.81) for liver conditions (Model 2, Table 2). After further adjusting for childhood abuse, the associations between any lifetime IPV and sight conditions, bronchitis, and arthritis were no longer significant for women (Model 3, Table 2).

For men, after adjusting for socioeconomic factors, exposure to any lifetime IPV was not associated with having at least one health condition. Regarding specific health conditions, exposure to any lifetime IPV was associated with an increased risk of having 4 out of 19 studied health conditions, including migraine, bowel/colon conditions, musculoskeletal conditions, and infectious diseases (Model 2, Table 3). The results for men no longer held significance for bowel/colon conditions and infectious diseases after further adjustment for childhood abuse (Model 3, Table 3).

### Lifetime physical IPV

A similar pattern to that reported for exposure to any lifetime IPV was observed for lifetime physical IPV. After adjusting for socioeconomic characteristics, women’s exposure to physical IPV was associated with increased odds of having at least one health condition (1.27, 1.03–1.57). Exposure to lifetime physical IPV was also associated with lower odds of experiencing good general health among women (0.53, 0.44–0.65).

Regarding associations with specific health conditions, women’s exposure to physical IPV was associated with increased odds of having 14 of the studied health conditions, including two additional conditions (diabetes and heart attack) that were not associated with any lifetime IPV. The full list included diabetes, migraine, heart attack, bronchitis, asthma, allergies, gastrointestinal conditions, arthritis, musculoskeletal conditions, infectious diseases, and conditions with liver, bowel/colon, bladder, and skin (Model 2, Table 2).

After adjustment for childhood abuse, the results remained significant for every health condition associated with physical IPV in Model 2, except for migraines, diabetes, and bronchitis (Model 3, Table 2).

Among men, after controlling for socioeconomic characteristics, no significant association was found between exposure to physical IPV and having at least one health condition. Bowel/colon conditions were the only health condition showing a significant association with exposure to physical IPV among men (Model 2, Table 3), but this association was no longer significant after adjustment for childhood abuse (Model 3, Table 3).

### Lifetime sexual IPV

After adjusting for socioeconomic factors, women’s exposure to lifetime sexual IPV was not significantly associated with having at least one physical health condition (1.46, 0.99–2.16). However, the odds of experiencing 9 specific health conditions, including migraines, sight conditions, asthma, allergies, and liver, bowel/colon, bladder, and musculoskeletal conditions, were higher for women who experienced lifetime sexual IPV compared to those who did not. The odds of experiencing good general health were also lower among women who reported experiencing lifetime sexual IPV (0.42, 0.29–0.62, Model 2, Table 2).

After further adjustment for childhood abuse, the associations between lifetime sexual IPV and the health conditions that were significant in Model 2 remained significant, with the only exception being skin problems, which were no longer significant in Model 3 (Table 2).

Similar to the patterns observed for women, exposure to lifetime sexual IPV was not associated with having at least one health condition in men. However, the experience of sexual IPV was associated with neurological conditions and bladder problems among men. These associations remained significant after adjustment for childhood abuse (Model 3, Table 3).

### Lifetime psychological IPV

For women, the associations between psychological IPV and health conditions were similar to those found for physical IPV. After adjusting for socioeconomic characteristics, a significant association was found between women’s exposure to lifetime psychological IPV and having at least one health condition (1.33, 1.09–1.64). Additionally, there was a negative association between exposure to psychological IPV and reporting good general health (0.49, 0.39–0.60).

Regarding specific health conditions, after adjusting for socioeconomic factors, significant associations were found between experiencing lifetime psychological IPV and each health outcome outlined for physical IPV, except for diabetes. This means there was a statistically significant association between experiencing psychological IPV and migraines, heart attack, bronchitis, asthma, allergies, gastrointestinal conditions, liver conditions, bowel/colon conditions, bladder problems, arthritis, musculoskeletal conditions, infectious diseases, and skin problems (Model 2, Table 2).

After adjusting for childhood abuse (Model 3, Table 2), the associations between psychological IPV and most health conditions in Model 2 for women remained significant, except for bronchitis and allergies.

For men, after controlling for socioeconomic factors, no significant association was found between ever experiencing psychological IPV and having at least one health condition. However, a negative association was found with good general health; that is, men who had ever experienced psychological IPV were less likely to report good general health (0.49, 0.39–0.60). Regarding specific health conditions, among men, experiencing lifetime psychological IPV was associated with higher odds of having 6 health conditions, namely neurological conditions, bowel/colon conditions, bladder conditions, arthritis, musculoskeletal conditions, and skin conditions (Model 2, Table 3). All of these associations remained significant after controlling for childhood abuse, except for bladder conditions (Model 3, Table 3).

### Lifetime economic IPV

After adjusting for socioeconomic characteristics, a significant association was found between women’s exposure to lifetime economic IPV and having at least one health condition (1.55, 1.21–1.99). Additionally, women exposed to economic IPV were less likely to report good general health (0.46, 0.36–0.61) (Model 2, Table 2).

Regarding specific health conditions, after adjusting for socioeconomic factors, significant associations were found between ever experiencing economic IPV and 10 of the studied health conditions, including migraines, high blood pressure, asthma, allergies, gastrointestinal conditions, bowel/colon conditions, arthritis, musculoskeletal conditions, infectious diseases, and skin conditions among women (Model 2, Table 2). After further adjustment for the experience of childhood abuse (Model 3, Table 2), the significant associations between economic IPV and most health conditions in Model 2 remained significant for women, except for allergies and musculoskeletal conditions.

For men, after controlling for socioeconomic factors, no significant association was found between ever experiencing economic IPV and having at least one health condition. However, a significant negative association was found with good general health; that is, men who had ever experienced economic IPV were less likely to report good general health (0.46, 0.36–0.61).

Regarding specific health conditions, among men, those who had ever experienced economic IPV were more likely to report having migraines, neurological conditions, allergies, and bowel/colon conditions in models adjusted for socioeconomic characteristics (Model 2, Table 3). All of these associations remained significant after controlling for childhood abuse, except for migraines and allergies (Model 3, Table 3).

### Any IPV in past 12 months

After adjusting for socioeconomic factors, women who experienced any type of IPV during the past 12 months had higher odds of having at least one health condition compared to women who did not experience any 12-month IPV (1.51, 1.10–2.09). Further adjustment for childhood abuse slightly attenuated the odds ratio, but it remained significant (1.46, 1.05–2.03). Additionally, exposure to any 12-month IPV was associated with lower odds of experiencing good general health among women (0.49, 0.36–0.67).

Regarding specific health conditions, after adjusting for socioeconomic factors, 11 out of 19 studied health conditions showed significant associations with experiencing any 12-month IPV among women. These conditions included migraines, hearing conditions, high blood pressure, asthma, allergies, gastrointestinal conditions, liver conditions, bowel/colon conditions, bladder conditions, musculoskeletal conditions, and skin conditions. The AORs ranged between 1.54 (1.06–2.25) for asthma and 7.48 (3.31–16.90) for liver conditions (Model 2, Table 2). After further adjustment for childhood abuse, the associations between 12-month IPV and hearing conditions, asthma, and gastrointestinal conditions were no longer significant for women (Model 3, Table 2).

For men, experiencing any IPV in the past 12 months was only associated with having infectious diseases, and this association remained significant even after controlling for childhood abuse (Models 2 and 3, Table 3).

### Multiple IPV forms

For women, after adjusting for socioeconomic characteristics, a cumulative pattern was observed, where an increase in the number of IPV types experienced was associated with an increased likelihood of physical health conditions. Women who experienced one type of IPV were more likely to report each of the 7 health conditions. This number increased to 8 for those exposed to two types of IPV and to 11 for those who experienced 3 or 4 types of IPV. The health conditions reported included migraine, sight conditions, asthma, allergies, gastrointestinal conditions, liver conditions, bowel/colon conditions, arthritis, musculoskeletal conditions, infectious diseases, and skin conditions. Additionally, as the number of IPV types increased, the odds of reporting good general health decreased. Women exposed to two types of IPV had a 44% decrease in the odds of experiencing good general health (0.56, 0.42–0.76), which further decreased to 68% for those exposed to three or four types of IPV (0.32, 0.24–0.42).

The experience of additional IPV types also appeared to increase the magnitude of the AORs for several conditions. For example, the AORs for musculoskeletal conditions ranged from 1.42 (1.13–1.80) for exposure to one IPV type and 1.47 (1.13–1.91) for exposure to two IPV types, to 1.74 (1.31–2.32) for exposure to three or four IPV types. The overall trend indicates a cumulative association between exposure to multiple IPV types and certain health conditions. However, not all health conditions showed a consistent incremental increase, and some associations did not reach statistical significance (Table 4). For men, after adjusting for socioeconomic characteristics, the likelihood of reporting health conditions increased with the number of IPV types experienced. Men who experienced one type of IPV were more likely to report one health condition compared to those with no exposure to IPV. This number increased to four health conditions for those exposed to two types of IPV and to six health conditions for those who experienced three or four types of IPV. However, no clear stepwise association was found between the number of IPV types experienced and any individual health condition (Table 5).

**Table 4.** Association between number of IPV types women experienced and current physical health conditions.

IPV count	1 IPV type		2 IPV types		3 or 4 IPV types	
Health condition	OR (95% CI)	AOR (95% CI)	OR (95% CI)	AOR (95% CI)	OR (95% CI)	AOR (95% CI)
Cancer	0.98 (0.46–2.07)	1.18 (0.56–2.51)	0.49 (0.17–1.46)	0.64 (0.21–1.98)	1.52 (0.69–3.31)	2.00 (0.91–4.40)
Diabetes	0.92 (0.58–1.44)	1.21 (0.75–1.96)	1.19 (0.74–1.94)	**1.74 (1.01–3.01)**	0.96 (0.56–1.65)	1.21 (0.68–2.14)
Migraine	1.25 (0.95–1.65)	1.15 (0.87–1.51)	**1.90 (1.44–2.50)**	**1.60 (1.19–2.15)**	**2.39 (1.79–3.18)**	**2.00 (1.47–2.70)**
Neurological conditions	1.31 (0.59–2.89)	1.73 (0.75–4.00)	0.39 (0.12–1.30)	0.44 (0.12–1.55)	2.13 (0.78–5.80)	1.94 (0.57–6.66)
Cataract/sight conditions	1.07 (0.79–1.44)	1.21 (0.89–1.66)	1.09 (0.82–1.45)	1.25 (0.90–1.73)	1.29 (0.92–1.82)	**1.48 (1.02–2.16)**
Hearing/ear conditions	1.02 (0.71–1.45)	1.33 (0.91–1.93)	1.07 (0.71–1.60)	1.34 (0.87–2.08)	0.96 (0.62–1.49)	1.16 (0.73–1.85)
Heart attack	0.84 (0.39–1.81)	1.20 (0.52–2.73)	1.22 (0.59–2.53)	2.05 (0.92–4.56)	1.48 (0.61–3.63)	1.84 (0.70–4.88)
High blood pressure	0.83 (0.62–1.12)	1.07 (0.78–1.48)	0.83 (0.61–1.11)	1.13 (0.82–1.57)	0.93 (0.67–1.29)	1.18 (0.81–1.73)
Bronchitis	1.65 (0.99–2.77)	**2.04 (1.17–3.55)**	1.18 (0.62–2.24)	1.54 (0.81–2.94)	1.64 (0.82–3.27)	1.98 (0.92–4.26)
Asthma	**1.37 (1.00–1.88)**	**1.39 (1.01–1.92)**	**1.45 (1.06–1.99)**	**1.43 (1.02–2.00)**	**2.60 (1.85–3.64)**	**2.40 (1.67–3.45)**
Allergies	**1.64 (1.24–2.18)**	**1.58 (1.18–2.11)**	1.25 (0.90–1.73)	1.13 (0.81–1.58)	**2.06 (1.50–2.82)**	**1.88 (1.34–2.62)**
Gastrointestinal conditions	**1.61 (1.12–2.31)**	**1.72 (1.19–2.50)**	**1.83 (1.27–2.64)**	**2.06 (1.38–3.06)**	**2.07 (1.33–3.22)**	**2.09 (1.31–3.34)**
Liver conditions	0.78 (0.22–2.83)	0.84 (0.23–3.04)	**3.63 (1.47–8.96)**	**3.77 (1.47–9.67)**	**5.36 (2.00–4.41)**	**5.52 (2.05–14.89)**
Bowel/colon conditions	1.24 (0.84–1.84)	1.31 (0.87–1.95)	**2.11 (1.49–3.00)**	**2.32 (1.59–3.39)**	**3.06 (2.11–4.44)**	**3.51 (2.34–5.24)**
Bladder conditions	**1.83 (1.21–2.77)**	**2.14 (1.40–3.27)**	**1.96 (1.27–3.04)**	**2.38 (1.47–3.85)**	**1.85 (1.14–3.00)**	**2.12 (1.28–3.53)**
Arthritis	0.81 (0.62–1.07)	1.04 (0.78–1.40)	0.92 (0.68–1.24)	1.36 (0.95–1.96)	**1.37 (1.03–1.83)**	**2.00 (1.39–2.86)**
Musculoskeletal conditions	**1.34 (1.07–1.68)**	**1.42 (1.13–1.80)**	**1.38 (1.08–1.77)**	**1.47 (1.13–1.91)**	**1.76 (1.35–2.31)**	**1.74 (1.31–2.32)**
Infectious disease	0.60 (0.07–5.08)	0.59 (0.07–5.14)	2.65 (0.63–11.23)	2.95 (0.59–14.8)	**9.72 (2.31–40.96)**	**9.57 (1.88–48.58)**
Skin conditions	**1.75 (1.31–2.35)**	**1.68 (1.25–2.27)**	**1.55 (1.12–2.13)**	1.39 (0.99–1.94)	**2.47 (1.75–3.49)**	**2.14 (1.49–3.09)**
Any chronic condition	0.96 (0.75–1.23)	1.17 (0.90–1.52)	1.02 (0.80–1.29)	1.30 (0.99–1.70)	**1.40 (1.06–1.87)**	**1.65 (1.20–2.27)**
Good general health	0.95 (0.73–1.23)	0.82 (0.63–1.08)	**0.63 (0.48–0.83)**	**0.56 (0.42–0.76)**	**0.33 (0.25–0.42)**	**0.32 (0.24–0.42)**

Abbreviations: AOR, adjusted odds ratio; CI, confidence interval; IPV, intimate partner violence; OR, odds ratio; Reference was no exposure (zero IPV).

Odds ratios adjusted for age, ethnicity, whether can keep home warm in winter, any serious debt, and area deprivation level.

Bold figures in the table indicate 95% CI significance at a two-sided *p* < 0.05.

**Table 5.** Association between the number of IPV types men experienced and current physical health conditions.

IPV count	1 IPV type		2 IPV types		3 or 4 IPV types	
Health condition	OR (95% CI)	AOR (95% CI)	OR (95% CI)	AOR (95% CI)	OR (95% CI)	AOR (95% CI)
Cancer	0.55 (0.20–1.52)	0.93 (0.38–2.29)	0.24 (0.03–1.83)	0.26 (0.04–1.86)	NO	NO
Diabetes	0.47 (0.27–0.81)	0.66 (0.37–1.17)	1.29 (0.65–2.56)	1.34 (0.63–2.87)	0.92 (0.31–2.71)	0.84 (0.22–3.27)
Migraine	**1.67 (1.08–2.57)**	1.44 (0.93–2.24)	1.86 (0.99–3.52)	1.34 (0.68–2.62)	**3.93 (1.79–8.61)**	**2.67 (1.16–6.17)**
Neurological conditions	1.63 (0.67–3.94)	1.78 (0.65–4.88)	1.66 (0.61–4.53)	1.05 (0.32–3.46)	**5.73 (1.50–21.9)**	**5.54 (1.23–24.9)**
Cataract/sight conditions	0.68 (0.47–0.99)	0.85 (0.58–1.24)	**1.67 (1.04–2.68)**	1.53 (0.92–2.55)	0.75 (0.26–2.17)	0.51 (0.15–1.71)
Hearing/ear conditions	0.85 (0.57–1.27)	1.20 (0.79–1.84)	0.65 (0.34–1.25)	0.71 (0.35–1.43)	0.58 (0.16–2.07)	0.72 (0.17–3.00)
Heart attack	0.62 (0.24–1.63)	0.87 (0.31–2.47)	2.26 (0.88–5.80)	1.69 (0.64–4.47)	0.60 (0.08–4.74)	0.66 (0.08–5.58)
High blood pressure	0.86 (0.62–1.20)	1.33 (0.93–1.91)	0.85 (0.50–1.44)	0.78 (0.45–1.35)	1.25 (0.51–3.08)	1.39 (0.47–4.06)
Bronchitis	0.28 (0.05–1.45)	0.36 (0.06–2.03)	**2.61 (1.02–6.68)**	2.53 (0.77–8.27)	0.49 (0.06–3.86)	0.41 (0.04–4.13)
Asthma	1.10 (0.64–1.87)	1.08 (0.62–1.87)	**1.87 (1.05–3.34)**	1.72 (0.96–3.08)	1.37 (0.45–4.24)	1.24 (0.40–3.89)
Allergies	1.21 (0.76–1.91)	1.17 (0.73–1.88)	1.45 (0.73–2.87)	1.47 (0.72–3.02)	**2.58 (1.10–6.05)**	**2.71 (1.14–6.45)**
Gastrointestinal conditions	1.15 (0.68–1.94)	1.27 (0.73–2.20)	1.77 (0.94–3.34)	1.75 (0.90–3.38)	1.93 (0.70–5.28)	1.68 (0.56–5.02)
Liver conditions	1.24 (0.44–3.52)	1.29 (0.43–3.86)	**5.01 (1.84–13.6)**	**2.98 (1.12–7.89)**	NO	NO
Bowel/colon conditions	0.89 (0.46–1.73)	1.09 (0.56–2.15)	1.68 (0.77–3.65)	2.11 (0.95–4.67)	**7.02 (3.03–16.3)**	**7.13 (2.79–18.2)**
Bladder conditions	0.47 (0.22–0.98)	0.68 (0.32–1.49)	**2.13 (1.09–4.17)**	**2.51 (1.27–4.95)**	0.83 (0.16–4.39)	1.31 (0.24–7.19)
Arthritis	0.62 (0.40–0.97)	0.91 (0.57–1.46)	**1.93 (1.14–3.27)**	**2.13 (1.13–4.00)**	1.28 (0.55–3.01)	1.44 (0.56–3.72)
Musculoskeletal conditions	**1.35 (1.00–1.82)**	**1.36 (1.01–1.84)**	1.52 (0.99–2.34)	1.31 (0.84–2.03)	**2.29 (1.15–4.54)**	1.86 (0.92–3.77)
Infectious disease	1.96 (0.44–8.66)	2.21 (0.81–6.09)	3.60 (0.91–14.2)	**3.99 (1.11–14.3)**	NO	NO
Skin conditions	1.19 (0.77–1.84)	1.26 (0.81–1.95)	1.29 (0.70–2.38)	1.26 (0.65–2.42)	**2.43 (1.06–5.59)**	**2.56 (1.08–6.06)**
Any chronic condition	0.83 (0.61–1.12)	1.11 (0.80–1.53)	1.02 (0.67–1.57)	0.91 (0.58–1.42)	1.19 (0.56–2.51)	1.17 (0.49–2.84)
Good general health	0.93 (0.66–1.30)	0.78 (0.54–1.13)	**0.58 (0.37–0.90)**	0.76 (0.48–1.20)	**0.36 (0.18–0.71)**	**0.42 (0.20–0.90)**

Abbreviations: AOR, adjusted odds ratio; CI, confidence interval; IPV, intimate partner violence; OR, odds ratio; NO, no observation, Reference was no exposure (zero IPV).

Odds ratios adjusted for age, ethnicity, whether can keep home warm in winter, any serious debt, and area deprivation level.

Bold figures in the table indicate 95% CI significance at a two-sided *p* < 0.05.

A similar pattern was observed for general health. Men who experienced three or four types of IPV were less likely to report good general health, but no clear stepwise association was found across the number of IPV types experienced (Table 5).

## Discussion

Using a robust national probability sample of women and men, this study analysed gender patterns in the associations between different types and number of types of IPV and various specific physical health conditions. The results provide new evidence of the compounding and varying effects of gender and IPV victimisation on the risk of experiencing poor health in England. Our findings highlight significant gender differences in both the prevalence of IPV and the associated health conditions. Women were more likely to experience any type and a higher number of types of IPV than men, with 7.2% of women and 3.8% of men reporting experiencing IPV (at least one type) in the past year. This is in line with the estimates reported by the Crime Survey for England and Wales, which found that 5.0% of adults (6.9% women and 3.0% men) aged 16 years and over experienced domestic abuse in the year ending March 2022.^43^

Women’s exposure to any lifetime and 12-month IPV were associated with an increased likelihood of reporting a range of health conditions (12 and 11 conditions respectively), while men’s exposure to any lifetime and 12-month IPV was associated with a limited number of health conditions (4 and 1 conditions, respectively).

Among women, specific IPV types had differential associations with health conditions. Physical IPV was associated with an increased likelihood of reporting the highest number of health conditions (14 conditions), followed by psychological IPV (12 conditions), economic IPV (10 conditions), and sexual IPV (9 conditions). However, specific IPV types were inconsistently associated with poor health outcomes among men. Men who reported exposure to psychological IPV had the greatest number of associations with the assessed health conditions (7 conditions), followed by men who reported exposure to economic IPV (4 conditions), and sexual and physical IPV (2 and 1 condition, respectively).

Additionally, a cumulative pattern was observed between the number of IPV types experienced and associations with health conditions, but only among women. While among men, exposure to increasing numbers of IPV types did not clearly show a stepwise association with health outcomes; exposure to 3 or 4 IPV types was associated with an increased likelihood of reporting 5 conditions, although the confidence intervals were wide. This finding is relevant and substantiates the gender patterns found in this study, as experiences of multiple IPV types were more prevalent among women with 60.5% of women who reported any lifetime IPV experiencing at least 2 IPV types, in contrast to 35.4% of men who reported any lifetime IPV.

Even after adjusting for socioeconomic factors and childhood abuse, many of these associations remained significant, underscoring the persistent impact of IPV on women’s health. However, the associations with some health conditions such as sight conditions and bronchitis were no longer significant after accounting for childhood abuse, suggesting that early life experiences may also play a role in these health conditions. In general, our findings support the assertions that IPV exposure is a gendered phenomenon, affecting the health of men and women differently and to varying degrees.^33,44,45^

Multiple factors may have contributed to the greater physical health impact of IPV on women compared to men. Most notably, the observed gender differences in physical health associations may reflect the distinct patterns and severity of IPV experiences between women and men. Women are also more likely to experience more types of IPV, which has been considered an indicator of the severity of violence in previous research.^6,10,43^ In the current study, women were more than three times as likely to experience multiple (⩾2) forms of IPV compared with men (16.2% versus 5.2%). This aligns with previous research showing that women face more severe forms of violence and experience prolonged and repeated IPV,^10^ which could lead to greater cumulative negative health effects.^29^

Additionally, while not explored in this study, women might find it more challenging than men to leave abusive relationships due to factors such as protecting children, fear of harm, economic dependence, lack of resources such as financial constraints, lack of support, and cultural and religious beliefs.^46,47^ These factors can prolong their experiences of violence and exacerbate its physical health consequences.

Compared with men, women are more likely to experience IPV types associated with worse health conditions, including physical and sexual violence.^48^ In our study, women were, respectively, twice and 19 times more likely to experience physical and sexual IPV than men. Exposure to these forms of IPV can lead to direct physical injuries, chronic pain, and long-term health issues such as reproductive conditions and sexually transmitted infections.^17^

Moreover, women are more likely to endure non-physical IPV types such as psychological abuse,^6,9,43^ a finding supported by data from the present study, which can result in long-term mental health issues like depression, anxiety, and post-traumatic stress disorder (PTSD).^9,49,50^ Similarly, a study using APMS data found evidence of association between psychological abuse in the form of receiving threatening/obscene messages and experience of common mental disorder, self-harm, and suicidal thoughts.^9^

The association of IPV (all forms) with poor mental health is well documented in the literature.^49^ It has also been suggested that resulting mental health conditions, particularly depression and PTSD, may mediate the pathway to poor physical health.^11,18,50,51^ While our cross-sectional study could not confirm causal pathways between IPV experiences and poor physical health, this remains an important avenue for future research. The psychological trauma from IPV can lead to chronic stress and prolonged physiological stress activity, which negatively affects physical health over time.^52^ Evidence also shows that the adoption of health risk behaviours and biological dysfunction may act as mediators in the link between IPV and poor health.^18,53^

Psychological IPV has been identified as the most common type of IPV experienced by men,^7^ a finding corroborated by data from the present study. However, in our study, exposure to psychological IPV was linked to 7 of the assessed health conditions in men, compared to 12 health conditions in women. This disparity may be due to differences in the co-occurrence of IPV types experienced by men and women.^7^ Women are more likely to experience psychological IPV in conjunction with other IPV types,^54^ leading to compounded health consequences, whereas men may not experience this co-occurrence as frequently. Additionally, men’s experiences of IPV may reflect infrequent or sporadic incidents, whereas women’s reports of IPV may reflect chronic patterns associated with worse health outcomes. The lower rates of multiple IPV types experienced by men compared to women in this study further support this claim.

Economic abuse, a less studied form of non-physical IPV, was also more prevalent among women than men in this study (8.7% versus 3.7%). Similarly, a large population survey in Australia found that women are more likely to be victims of economic abuse compared to men.^55^ Economic IPV encompasses behaviours that control a survivor’s ability to acquire, use, and maintain resources, potentially leading to economic dependence on their partner. This dependence may limit their ability to leave the relationship and establish independence which could lead to prolonged exposure to IPV and contribute to poor health outcomes, particularly among women.^46,56^

Of note, in this study, women were more likely to have limited financial resources, residing in moderately or highly deprived areas and struggling to keep their homes warm during winter. This financial constraint can restrict access to healthcare and social support services, exacerbating the health impacts of IPV. Social and cultural factors such as cultural norms and stigma surrounding IPV (while not explored in this study) can also discourage women from seeking help or reporting abuse,^57,58^ resulting in untreated health conditions and exacerbating poor health outcomes.^59–61^

Our findings highlight the long-term physical health implications of IPV, positioning it alongside other critical social determinants of health such as socioeconomic status, education, and access to healthcare, especially for women. To effectively address the heightened risk of adverse health outcomes linked to women’s exposure to IPV, health professionals must develop a nuanced understanding of IPV identification and responses.^62,63^ They should also be supported with strong referral options within proactive and dynamic healthcare systems. Creating these responsive healthcare systems requires well-designed and comprehensive IPV curricula in medical and health training programs.^63^

Although we did not assess the precise overlap of specific IPV types, existing literature indicates that psychological/emotional IPV is the most commonly reported standalone type of IPV, while physical and sexual IPV frequently co-occur with psychological IPV.^64^ Future research should explore the interplay between different types of IPV in greater detail, examining how these overlapping experiences affect health outcomes across gender groups.

### Strengths

Our study addresses several key gaps in the existing literature and provides novel insights into gendered associations between IPV and physical health. By utilising data from a representative probability sample in England, we offer a more generalisable perspective compared to studies that rely on participants from IPV support or healthcare services. Our research uniquely examines gender differences in the associations between both lifetime and recent (past 12-month) IPV exposure, disaggregated by type and cumulative exposure, revealing significant disparities in health outcomes between men and women. Unlike previous studies that have predominantly focused on mental health impacts or aggregated physical health conditions, our study provides a detailed analysis of multiple specific physical health conditions, including those that have been less thoroughly explored such as cardiovascular and neurological conditions. Additionally, our research highlights the interplay between different forms of IPV, including less visible psychological and economic abuse, and their cumulative effects on health, which is often overlooked in the literature. By addressing these dimensions, our findings offer a more comprehensive understanding of how IPV impacts health and underscore the need for targeted interventions.

### Limitations and future directions

This study has limitations. While the data utilised were the most current available at the time of this research, they are a decade old. Although this temporal gap may raise concerns, we do not anticipate that the fundamental nature of the associations between IPV and health outcomes has significantly changed during this period. However, it is important to acknowledge that we were unable to account for the potential impact of more recent factors, such as the COVID-19 pandemic, which may have influenced both IPV dynamics and health outcomes in ways that our data cannot capture. Sampling factors may have contributed to an underestimation of the prevalence of IPV and health conditions, including the exclusion of those living in inaccessible housing, people who could not speak English, and residents of facilities such as prisons, hospitals, and residential homes. Those who were unwell may have also been less likely to participate. These exclusions may have potentially skewed the sample towards relatively healthy individuals.

People recently exposed to IPV might have been less likely to participate, particularly those subject to current coercive control from a partner, which may have made them less able to take part in a research study. This could potentially introduce selection bias. Reasons for non-disclosure may differ by gender; for instance, men may underreport IPV due to stereotypes and fear of ridicule.^65^ Women, meanwhile, may withhold disclosure of IPV experiences due to fears of retaliation, stigma, or feelings of shame, which could be heightened if they are experiencing ongoing abuse.^66–68^ Additionally, recall or social desirability biases could have affected self-reported measures of IPV exposure in both men and women.^69,70^

The use of a single-item measure for economic abuse and a two-item measure for psychological IPV may have limited the assessment to a narrow range of behaviours, potentially overlooking other forms that participants may have experienced. This brief approach could result in underestimating both the prevalence and impact of these types of IPV, as it may misclassify individuals who experienced unlisted behaviours as not having experienced this type of abuse. This highlights the need for more comprehensive and nuanced measures in future research. Similarly, our study’s focus on physical and sexual abuse in childhood, without addressing emotional abuse, maltreatment, or neglect, represents a limitation.

A few point estimates of different types of IPV may be imprecise due to wide CIs, especially for men. Furthermore, while our study identifies associations between specific IPV types and health outcomes, the cross-sectional nature of our data limits our ability to confirm causal pathways between IPV and poor physical health. However, longitudinal studies have explored these relationships in more depth, offering insights into the potential causal effects of IPV on physical health outcomes over time.^64,71^ Moreover, our study did not consider sexual orientation nor identification as with a non-binary gender. Due to limitations in sample size, our study was unable to stratify analyses by socioeconomic groups, which may obscure socioeconomic-specific differences in the IPV-health relationship. As a secondary analysis, the sample size was not determined by the needs of the current analysis and thus is relatively underpowered. Addressing this gap in future studies could reveal important nuances in how IPV impacts health across different demographic groups, which is crucial for developing inclusive interventions and support systems that meet the diverse needs of survivors.

Although we accounted for key confounders, limitations in sample size and the need to avoid over-adjustment – particularly for factors that may be part of the causal pathway, such as health-related behaviours – restricted our ability to include all potential confounders. This may introduce residual confounding, potentially affecting the interpretation of our findings within the diverse context of British society.

Future research should explore the specific pathways linking IPV to health outcomes, with a particular emphasis on mechanisms affecting men, as much less is known about these mechanisms among men compared to women. This would help build a more comprehensive understanding of IPV’s health impacts across genders.

Additionally, future research should explore the potential bidirectional nature of the relationship between IPV and health. For instance, while physical trauma from IPV can lead to visual impairments or sight loss, those with pre-existing visual impairments may be more vulnerable to IPV due to their reduced capacity to avoid or respond to threats.^72,73^

## Conclusion

Overall, utilising a robust national probability sample, rigorous data collection methods, and a purposefully designed analysis to address underexplored IPV types and specific health outcomes, our finding further substantiates claims that experience of IPV and its health impacts are gendered phenomena. Women are not only more likely to experience various forms of IPV, but their exposure also translates to a higher risk of numerous chronic health conditions. The intersection of multifaceted factors, including the frequency and severity of IPV, types of violence experienced, socioeconomic conditions, social and cultural influences, and psychological impacts, may have contributed to the greater health impact of IPV on women compared to men observed in this study. The observed gendered pattern highlights the need for targeted interventions and support systems that address the specific health needs of IPV survivors, with a particular focus on the compounded health risks faced by women.

## Supplemental Material

sj-docx-1-whe-10.1177_17455057251326419 – Supplemental material for Intimate partner violence and physical health in England: Gender stratified analyses of a probability sample survey Supplemental material, sj-docx-1-whe-10.1177_17455057251326419 for Intimate partner violence and physical health in England: Gender stratified analyses of a probability sample survey by Ladan Hashemi, Anastasia Fadeeva, Nadia Khan and Sally McManus in Women’s Health

sj-docx-2-whe-10.1177_17455057251326419 – Supplemental material for Intimate partner violence and physical health in England: Gender stratified analyses of a probability sample survey Supplemental material, sj-docx-2-whe-10.1177_17455057251326419 for Intimate partner violence and physical health in England: Gender stratified analyses of a probability sample survey by Ladan Hashemi, Anastasia Fadeeva, Nadia Khan and Sally McManus in Women’s Health

## Declarations

**Ethics approval and consent to participate:** The secondary data analyses were approved by the committee at City St George’s, University of London that considers medium-risk applications (ETH21220–299). The authors assert that all procedures contributing to this work comply with the ethical standards of the relevant national and institutional committees on human experimentation and with the Helsinki Declaration of 1975, as revised in 2008. Permission to use the data was granted by NHS England.

**Availability of data and materials:** The APMS dataset, data dictionary, protocol, participant materials, and full documentation are lodged with the UK Data Service. Permission to use the dataset for this analysis was obtained from NHS England. Requests for further use should be made to the UK Data Service.
