# Supplementary material for: Intimate partner violence and physical health in England: Gender stratified analyses of a probability sample survey
Source: Womens Health (Lond). 2025 Mar 25;21:17455057251326419. doi: 10.1177/17455057251326419 (PMC11946296; doi:10.1177/17455057251326419)
Supplement: sj-docx-2-whe-10.1177_17455057251326419 – Supplemental material for Intimate partner violence and physical health in England: Gender stratified analyses of a probability sample survey [file sj-docx-2-whe-10.1177_17455057251326419.docx]

**Supplementary Table 1. Variable definition**

| IPV |  |
| --- | --- |
| Physical | Physical IPV was indicated if any of the following were endorsed: “pushed you, held or pinned you down, or slapped you” or “kicked you, bit you, or hit you with a fist or something else, or threw something at you that hurt you”, “been injured (even if only slightly) as a result of the force used on you? **^*^**(By injured we mean things such as bruises, black eyes, cuts or scratches, or broken bones). |
| Sexual | Sexual IPV was recorded if either of the following were endorsed: “since the age of 16, has anyone touched you, or got you to touch them, in a sexual way without your consent?” or “since the age of 16, has anyone had sexual intercourse with you without your consent?”, or both, and if the perpetrator was a current or former partner. |
| Psychological | Psychological IPV was identified where either of the following were endorsed: “repeatedly belittled you to the extent that you felt worthless” or “sent you more than one unwanted letter, email, text message, or card that was either obscene or threatening and which caused you fear, alarm, or distress”. |
| Economic | Economic IPV was recorded if the participant reported that a partner had “prevented you from having your fair share of the household money”. |
| Physical health outcomes | Have you had any of these health conditions in the past 12 months?  1 Cancer  2 Diabetes  3 Migraine or frequent headaches  4 Neurological problems including dementia or Alzheimer's disease, epilepsy/fits, and stroke  5 Cataracts/eyesight problems (even if corrected with glasses or contacts)  6 Ear/hearing problems (even if corrected with a hearing aid)  7 Heart attack/angina  8 High blood pressure  9 Bronchitis/emphysema  10 Asthma  11 Allergies  12 Gastrointestinal problems including digestive problems and stomach ulcer  13 Liver problems  14 Bowel/colon problems  15 Bladder problems/incontinence  16 Arthritis  17 Musculoskeletal problems including bone, back, joint or muscle problems  18 Infectious disease  19 Skin problems |
| Good general health | Having a good health was recorded if the participant reported that their heath in general was “Excellent”, “Very good”, or “Good”. “Poor or fair” responses were recorded as not having a good general health. |
| Child abuse | Child abuse was identified if any of the following were endorsed: “Before the age of 16, did anyone touch you, or get you to touch them, in a sexual way without your consent?” or “Before the age of 16, did anyone have sexual intercourse with you without your consent?”, or “Not including smacking, before you were 18, did an adult in your life hit, beat, kick, or physically hurt you in any way?”. |
